# Proteomic characteristics of bronchoalveolar lavage fluid in children with mild and severe *Mycoplasma pneumoniae* pneumonia

**DOI:** 10.3389/fmicb.2025.1595521

**Published:** 2025-05-19

**Authors:** Ao Liang, Yaqi Zhu, Xiaoxue Wu, Qingyan Zhang, Yafang He, Anbang Wang, Chunchen Wu, Jianbo Xia

**Affiliations:** ^1^Department of Laboratory Medicine, Maternal and Child Health Hospital of Hubei Province, Tongji Medical College, Huazhong University of Science and Technology, Wuhan, China; ^2^Department of Pediatrics, Maternal and Child Health Hospital of Hubei Province, Tongji Medical College, Huazhong University of Science and Technology, Wuhan, China; ^3^SpecAlly Life Technology Co., Ltd., Wuhan, China; ^4^School of Laboratory Medicine, Hubei University of Chinese Medicine, Wuhan, China; ^5^Hubei Provincial Center for the Prevention and Treatment of Pediatric Infectious Diseases, Wuhan, China

**Keywords:** *Mycoplasma pneumoniae* pneumonia, quantitative proteomics, differentially expressed proteins, bronchoalveolar lavage fluid, predictive model

## Abstract

**Objectives:**

*Mycoplasma pneumoniae* pneumonia (MPP), particularly macrolide-resistant MPP has undergone a prolonged nonseasonal epidemic in China since the lifting of non-pharmaceutical interventions in 2023. This study aimed to identify novel biomarkers to predict disease severity in children with MPP and to develop a predictive model.

**Methods:**

In this study, bronchoalveolar lavage fluid (BALF) samples were collected from 30 children, including 15 with mild and 15 with severe MPP, for quantitative proteomic analysis. The two groups were compared and differentially expressed proteins (DEPs) were identified. Core proteins associated with MPP severity were identified using least absolute shrinkage and selection operator (LASSO) analysis. Logistic regression analysis was used to develop a predictive model.

**Results:**

A total of 154 DEPs were identified, of which 57 were upregulated in the severe group. Upregulated signaling was found to be mainly involved in the immune response and inflammatory signaling. Thirteen proteins were selected as core proteins associated with MPP severity. CD209, CHM, PBRM1, and SCAMP1 were the most influential predictors and a predictive model using these four proteins predicted MPP severity.

**Conclusion:**

A predictive model was developed to assess the potential of using the identified biomarkers to predict disease severity. This model provides insights into the pathogenesis of *M. pneumoniae* infection.

**Importance:**

Differences in the proteomic characteristics of bronchoalveolar lavage fluid in children with mild and severe *Mycoplasma pneumoniae* pneumonia (MPP) were identified, revealing the role of lung-specific immunologic and inflammatory response in the pathogenesis of MPP. Given the increasing incidence of severe MPP in children in recent years and the emergence of macrolide-resistant *M. pneumoniae* infection in some regions, our findings provided valuable knowledge and insights into the pathogenesis of *M. pneumoniae* infection.

## Introduction

*Mycoplasma pneumoniae* (*M. pneumoniae*) is a common cause of respiratory tract infections in children and is associated with a high burden of disease, with epidemics occurring every 3–7 years, accounting for approximately 10–30% of children with community-acquired pneumonia (CAP) (Jain et al., 2015). The most recent epidemic of *M. pneumoniae* occurred in late 2019 and early 2020 affecting multiple countries, predominantly in Europe and Asia, simultaneously (Meyer Sauteur et al., 2022). The introduction of non-pharmaceutical interventions (NPIs) against SARS-CoV-2 infection in 2020 resulted in a substantial reduction in the incidence of disease caused by many respiratory pathogens, including *M. pneumoniae* (Meyer Sauteur et al., 2022; Brueggemann et al., 2021; Xia et al., 2023). As NPIs were relaxed, a resurgence of respiratory infectious diseases was reported in multiple countries (Torres et al., 2023; Nygaard et al., 2023). However, compared with other respiratory pathogens, including *Mycobacterium tuberculosis,* the re-emergence of *M. pneumoniae* in Europe and Asia was delayed (Meyer Sauteur and Beeton, 2024). In China, the relaxation of COVID-19 NPIs triggered a nationwide *M. pneumoniae* epidemic that deviated from its typical seasonal pattern. This outbreak began in early 2023, intensified with school re-opening in September, and peaked in October and November 2023. This nonseasonal surge is attributable to reduced population immunity as a result of NPIs during the COVID-19 pandemic, and the dominance of macrolide-resistant genotypes (Li et al., 2024; Yan et al., 2024; Xu et al., 2024; Zhang et al., 2024). Compared with adults, children are more likely to develop pneumonia or severe disease (Edelstein et al., 2023). Additionally, the incidence rate of macrolide-resistant *M. pneumoniae* infection is higher in children than in adults, which complicates the clinical treatment of *M. pneumoniae* pneumonia (MPP) (Li et al., 2024; Akashi et al., 2018).

Current approaches to assessing MPP severity rely primarily on clinical signs, imaging findings, and the levels of nonspecific inflammatory markers such as C-reactive protein and lactate dehydrogenase (Zhang et al., 2024; Li et al., 2024). However, these methods lack sensitivity to predict early progression to severe disease and offer limited mechanistic insights into the host-pathogen interaction. Recent studies have explored candidate biomarkers such as interleukin 6 and tumor necrosis factor alpha (TNF-α) levels in serum or nasopharyngeal samples, but these focus on single proteins or narrow inflammatory pathways (Nagoba et al., 2024; Xu et al., 2016; Tian et al., 2020). Therefore, an understanding of the development and pathogenesis of MPP through studying bronchoalveolar lavage fluid (BALF) is important for the control and treatment of *M. pneumoniae* infections.

Proteomics research based on liquid chromatography–tandem mass spectrometry (LC–MS/MS) is playing an increasingly important role in understanding pathogenesis, diagnosis, and identification of drug and vaccine targets of infectious diseases (Shama et al., 2023). To address the lack of tools for prediction MPP severity, we used four-dimensional (4D)-DIA proteomics on BALF—a direct reflection of lung pathology—combined with machine learning. This approach identified a four-protein biomarker panel that achieved good results in predicting which patients would develop severe MPP, significantly outperforming traditional clinical parameters (Qiu-Ju et al., 2024; Chen et al., 2024). To our knowledge, this is the first study to integrate BALF proteomics with predictive modeling for predicting pediatric MPP severity, offering a paradigm shift from reactive clinical assessment to proactive molecular risk stratification.

In this study, BALF samples were collected from children with mild or severe MPP for quantitative proteomic analysis. This study identified differences in protein abundance biological processes, and disease pathways according to disease severity, based on differentially expressed proteins (DEPs) in the BALF. Furthermore, by leveraging machine learning, we developed a predictive model that integrates the results of multiple DEPs. This predictive model showed superior diagnostic performance compared with that of conventional clinical parameters. The four-protein biomarker panel also provides a tool for early risk stratification before clinical deterioration. This model could guide clinicians in initiating timely interventions (such as corticosteroid therapy or advanced respiratory support) for high-risk patients, while avoiding overtreatment in mild cases. Furthermore, the identified pathways (such as cytokine-cytokine receptor interaction) highlight potential therapeutic targets for modulating the host response in patients with severe MPP. By linking proteomic discoveries to practical clinical decision-making, this study lays the foundation for precision medicine in pediatric respiratory infections.

## Patients and methods

### Study design participants

The study included patients with MPP hospitalized in the Maternal and Child Health Hospital of Hubei Province between December 2023 and February 2024. The study was approved by the Research Ethics Committee of Maternal and Child Health Hospital of Hubei Province (2024-041-01). Each child’s parent or guardian provided informed consent prior to enrolment.

MPP was diagnosed as follows: (1) The main clinical manifestations were fever and cough, with or without headache, runny nose, sore throat, earache, and wheezing, particularly in infants and young children; (2) The imaging findings were characterized by capillary bronchitis, bronchopneumonia, and interstitial pneumonia; combined with one or more of the following laboratory criteria: (i) MP antibody titer (PA method) in single serum ≥1:160; or a greater than or equal to fourfold increase in the MP antibody titer during the course of the disease; (ii) MP-RNA positivity on nucleic acid amplification tests.

Severe MPP was defined as follows: (1) continuous high fever (above 39°C) for ≥5 days or fever for ≥7 days; (2) development of wheezing, shortness of breath, dyspnea, chest pain, or hemoptysis; (3) the presence of extrapulmonary complications; (4) pulse oxygen saturation ≤0.93 at rest, breathing room air; (5) imaging findings characterized by at least one of the following: uniform and consistent high-density consolidation of ≥2/3 of a single lobe, high-density consolidation of two or more lobes with a moderate to large pleural effusion or with localized bronchitis, diffuse capillary bronchitis in one lung, or capillary bronchitis of ≥4/5 lobes in both lungs, combined with bronchitis, and atelectasis resulting from the formation of mucous emboli; (6) progressively aggravated clinical symptoms, with extension of the lesion range by more than 50% in 24–48 h based on imaging; or (7) an obvious increase in C-reactive protein (CRP), lactate dehydrogenase (LDH), or D-dimer levels. Patients with immunodeficiency and those taking immunosuppressants were excluded.

The study was approved by the Ethics Committee of Maternal and Child Health Hospital of Hubei Province (2024-041-01). Each child’s parent or guardian provided written informed consent prior to enrolment.

### Bronchoalveolar lavage fluid sample preparation

BALF samples were collected from December 1, 2023, to February 29, 2024. The samples were prepared as described previously (Yuan et al., 2023). Briefly, the samples were mixed with SDC/Tris–HCl/TCEP/CAA solution to complete protein reduction and alkylation. Protein concentration was quantified by bicinchoninic acid assay (BCA), followed by trypsin digestion at 37°C overnight. Finally, the digested peptides were further purified and stored at −20°C.

### Liquid chromatography–tandem mass spectrometry and data processing

All Samples were analyzed using a hybrid timsTOF Pro mass spectrometer (Bruker Daltonics, Bremen, Germany) coupled with UltiMate 3000 RSLCnano (Thermo Fisher Scientific, Waltham, MA, United States). Mobile phases contained (i) 0.1% formic acid in water, and (ii) acetonitrile at 300 nL/min. MS was performed in diaPASEF mode with 1,400 V capillary voltage, scanning 100–1,700 m/z, with ion mobility at 0.6–1.6 *Vs*/cm^2^, and accumulation/ramp times of 100 ms. The diaPASEF acquisition windows were configured using timsControl software based on the m/z-ion mobility correlation pattern: the collision energy was linearly ramped from 59 eV to 20 eV.

The raw data from MS were processed using DIA-NN software (1.8.1) and matched to the *Homo sapiens* protein sequence database downloaded from UniProt (20230619) using the parameters as described previously (Wang et al., 2025).

### Protein functional annotation and the enrichment analyses

The Gene Ontology (GO)^1^ and Kyoto Encyclopedia of Genes and Genomes (KEGG)^2^ databases were used to assess protein function. GO and KEGG annotation information was obtained by aligning the identified proteins against the corresponding reference datasets retrieved from the official sources.

### Construction of the predictive model

The XGBoost machine-learning model was used to construct the predictive model. A total of 30 samples were randomly divided into a training set and a test set in a 4:1 ratio, with 24 samples in the training set, and 6 samples in the test set. In the training set, the LASSO regression model with five-fold cross-validation was used to screen DEPs. An optimal diagnostic model was constructed based on the area under the curve (AUC) and the calibration index (Brier score).

### Statistical analysis

The statistical analysis was performed as described previously (Wu et al., 2025). Participants’ demographic and clinical characteristics were described using the mean and standard deviation or the median and interquartile range for continuous variables with normal or non-normal distributions, respectively. The significance of differences in DEPs between children with mild and severe MPP was determined using Student’s *t*-tests. Proteins with a log2-fold change in abundance ≥0.58 and *p* < 0.05 were considered differentially abundant. The model development were performed using the R programming language (The R Foundation for Statistical Computing, Vienna, Austria).

## Results

### Study design and patients

**Figure 1:**
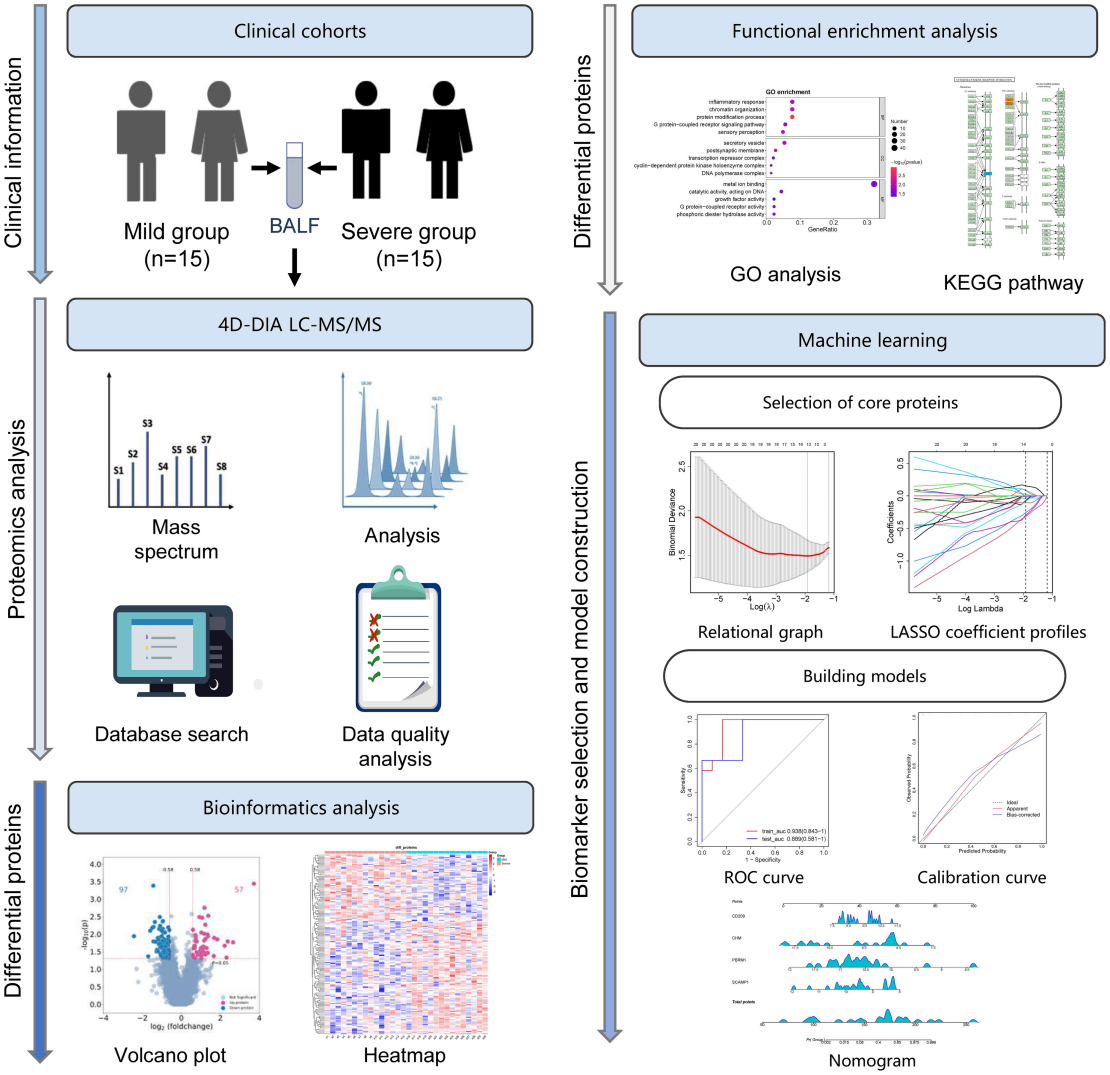
Study flowchart. Thirty children with *M. pneumoniae* pneumonia (MPP), including 15 with mild MPP and 15 with severe MPP, were enrolled in this study. Bronchoalveolar lavage fluid (BALF) samples from these patients were subjected to four-dimensional data-independent acquisition (4D-DIA) liquid chromatography–tandem mass spectrometry (LC–MS/MS) analysis. The differentially expressed proteins (DEPs) were identified and compared using bioinformatics analysis, followed by functional enrichment analysis including Gene Ontology (GO) and Kyoto Encyclopedia of Genes and Genomes (KEGG) pathway enrichment analysis. Finally, by machine learning, a series of core proteins were selected, and a prediction model was constructed and evaluated using the least absolute shrinkage and selection operator (LASSO) analysis and receiver operating characteristic (ROC) curves.

Thirty children with MPP, including 15 with mild MPP and 15 with severe MPP, were enrolled in this study (Figure 1). The BALF samples from these patients were subjected to 4D-DIA LC–MS/MS analysis. DEPs were identified and compared using bioinformatics analysis, followed by functional enrichment analysis, including GO and KEGG pathway enrichment analysis. Finally, core proteins were selected using machine learning and a prediction model was constructed and evaluated (Figure 1).

As shown in Table 1, patients in the mild and severe disease groups did not differ significantly (Table 1). The severe disease group had a significantly higher white blood cell counts and significantly lower TNF-α levels than those in the mild group. None of the other clinical indicators, including the lymphocyte count, neutrophil count, or hypersensitive CRP (hs-CRP), LDH, D-dimer, aspartate aminotransferase (AST), or alanine aminotransferase (ALT) levels, differed significantly between the mild and severe groups (Table 1).

**Table 1:** Demographic and clinical characteristics of the participants.

	Mild MPP (*n* = 15)	Severe MPP (*n* = 15)	*P* value^a^
**Demographic characters**			
Sex, *n* (%)			>0.999
Male	5 (33)	5 (33)	
Female	10 (67)	10 (67)	
Age (years), mean ± SD	7.9 ± 1.7	6.3 ± 2.8	0.079
**Hematology** ^a^			
White blood cells (×10^9^/L), mean ± SD	6.33 ± 1.28	10.54 ± 3.41	<0.001***
Lymphocytes (×10^9^/L), median (IQR)	1.53 (1.34, 1.71)	2.27 (1.43, 4.28)	0.061
Neutrophils (×10^9^/L), mean ± SD	4.19 ± 1.12	5.44 ± 3.06	0.168
**Inflammatory indicators**			
hs-CRP^b^ (mg/L), median (IQR)	12.01 (7.28, 17.90)	10.43 (1.87, 18.25)	0.560
LDH^c^ (U/L), mean ± SD	287.02 ± 42.99	308 ± 86.92	0.440
D-dimer^d^ (μg/mL), mean ± SD	0.47 ± 0.21	0.65 ± 0.32	0.090
AST^e^ (U/L), mean ± SD	26.56 ± 3.84	28.07 ± 8.66	0.569
ALT^e^ (U/L), mean ± SD	11.49 ± 4.33	14.67 ± 8.28	0.226
**Cytokines** ^f^			
IL-2, median (IQR)	4.95 (1.54, 5.85)	1.15 (0.81, 4.82)	0.315
IL-4, median (IQR)	3.14 (1.77, 3.61)	1.54 (1.11, 2.84)	0.055
IL-6, median (IQR)	9.86 (7.37, 13.38)	20.13 (4.38, 30.04)	0.515
IL-10, median (IQR)	8.56 (3.15, 12.37)	4.07 (2.75, 6.99)	0.360
TNF-α, median (IQR)	3.18 (2.72, 6.12)	2.14 (1.58, 2.62)	0.012*
IFN-γ, median (IQR)	2.90 (2.04, 7.72)	2.25 (1.58, 4.18)	0.237

a *n* = 14 in the mild group and *n* = 13 in the severe group.

b *n* = 14 in the mild group and *n* = 12 in the severe group.

c *n* = 13 in the mild group and *n* = 14 in the severe group.

d *n* = 14 in the mild group and *n* = 15 in the severe group.

e *n* = 13 in the mild group and *n* = 15 in the severe group.

f *n* = 8 in the mild group and *n* = 10 in the severe group.

ALT, alanine aminotransferase AST, aspartate aminotransferase; hs-CRP, hypersensitive C-reactive protein; LDH, lactic dehydrogenase. *p < 0.05; ***p < 0.001.

### Proteomic alterations in the BALF of children with mild and severe MPP

**Figure 2:**
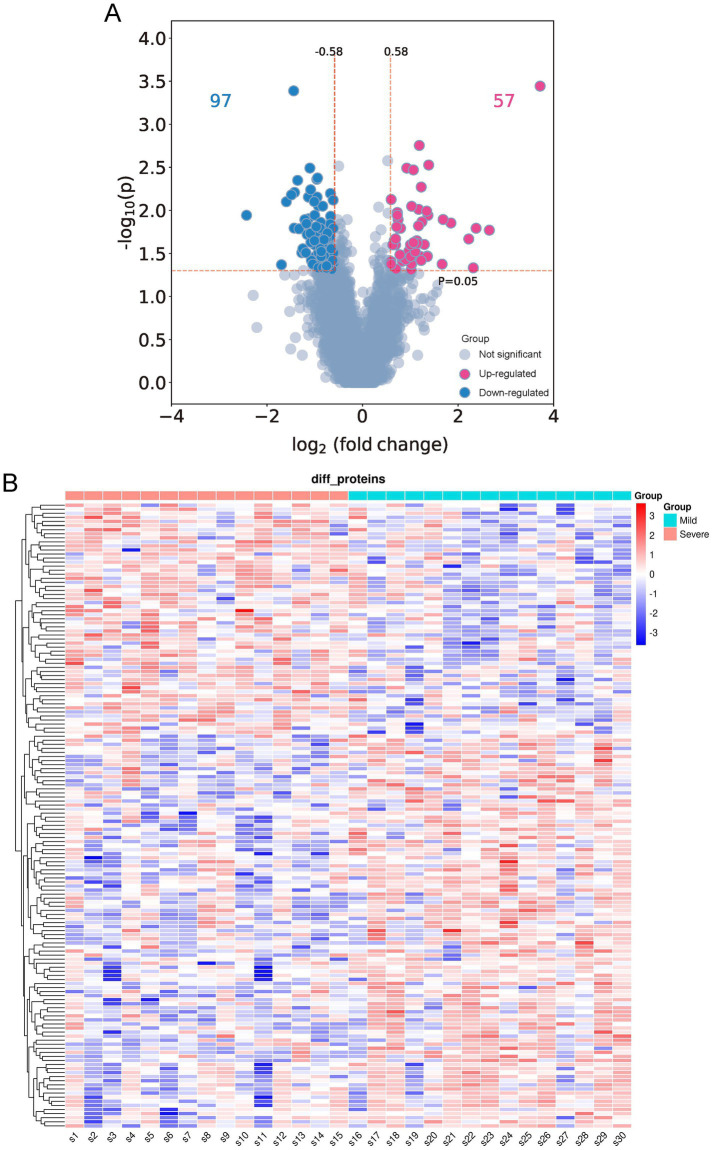
Differentially expressed proteins (DEPs) in the bronchoalveolar lavage fluid (BALF) of children with mild and severe *Mycoplasma pneumoniae* pneumonia (MPP). **(A)** Volcano plot of differentially expressed proteins (DEPs). A total of 154 DEPs (|log2-fold change| ≥ 0.58 and *p* < 0.05) were identified, of which 97 were downregulated (indicated with blue dots), and 57 were upregulated (indicated with red dots). **(B)** Quantitative heat maps of 154 DEPs: S1–S15, children with mild MPP; S16–S30, children with severe MPP.

A DIA strategy was used to investigate the differences in BALF proteome profiles between children in the severe and mild MPP groups. Thirty proteomes were examined in the entire cohort, yielding a total of 6,418 proteins and 45,150 precursors. The quality control results of the proteomics analyses indicated that the samples had good repeatability, and the principal components analysis (PCA) revealed that the DEPs in the severe group were clearly distinguishable from those in the mild group (Supplementary Figures S1A–D). A total of 154 proteins were significantly differentially expressed in patients with severe MPP compared with those with mild MPP, of which 57 proteins were upregulated, and 97 proteins were downregulated in patients with severe MPP (Figure 2A). A scaled heatmap was constructed to visually compare the severe with mild MPP groups (Figure 2B). The heatmap showed distinct proteomic patterns in the two groups.

### Functional enrichment analysis of differentially expressed proteins

**Figure 3:**
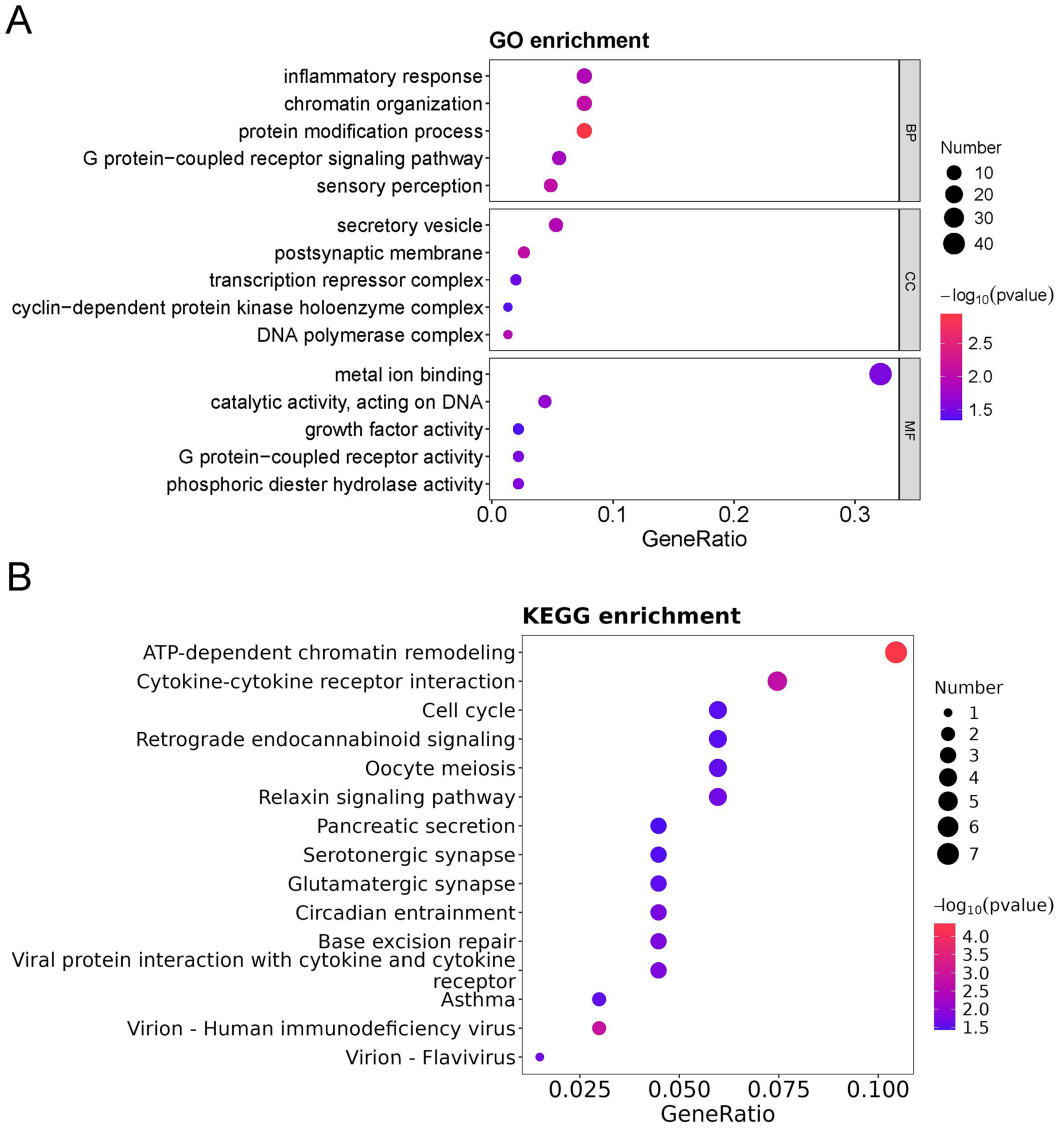
GO, KEGG, and DO analysis of all differentially expressed proteins (DEPs) in the bronchoalveolar lavage fluid (BALF) of children with mild and severe *Mycoplasma pneumoniae* pneumonia (MPP). **(A)** GO-based enrichment analysis of DEPs (two-sided hypergeometric test, *p* < 0.05), GO terms were sorted by *p* value, and the top 5 terms of each category are displayed. **(B)** KEGG-based enrichment analysis of DEPs (two-sided hypergeometric test, *p* < 0.05), KEGG terms were sorted by *p* value, and the top 15 terms are displayed.

To further characterize the functional identity of the 154 DEPs, GO and KEGG pathway analyses were performed. The top five annotations of the DEPs enriched in biological process (BP) were inflammatory response, chromatin organization, protein modification process, G protein-coupled receptor signaling pathway, and sensory perception. The top five annotations of DEPs enriched in molecular function (MF) were metal ion binding, catalytic activity acting on DNA, growth factor activity, G protein-coupled receptor activity, and phosphoric diester hydrolase activity. The top five annotations of DEPs enriched in the cellular component (CC) were secretory vesicle, postsynaptic membrane, transcription repressor complex, cyclin-dependent protein kinase holoenzyme complex, and DNA polymerase complex (Figure 3A). ATP-dependent chromatin remodeling and cytokine-cytokine receptor interaction were the two most significant signaling pathways (Figure 3B).

**Figure 4:**
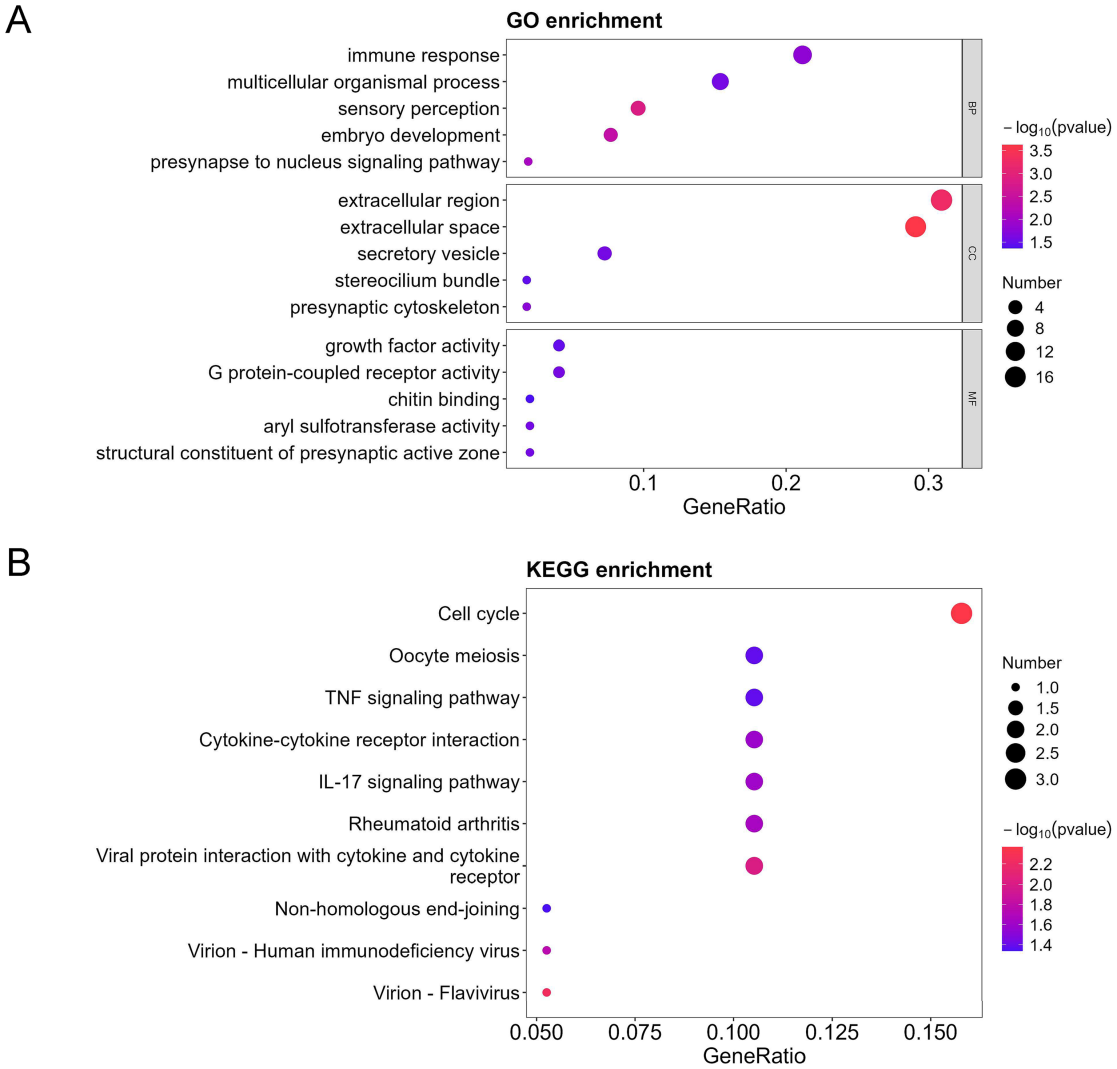
GO, KEGG, and DO analysis of the upregulated differentially expressed proteins (DEPs) in the bronchoalveolar lavage fluid (BALF) of children with mild and severe *Mycoplasma pneumoniae* pneumonia (MPP). **(A)** GO-based enrichment analysis of DEPs (two-sided hypergeometric test, *p* < 0.05), GO terms were sorted by *p* value, and the top 5 terms of each category are displayed. **(B)** KEGG-based enrichment analysis of DEPs (two-sided hypergeometric test, *p* < 0.05), KEGG terms were sorted by *p* value, and the top 15 terms are displayed.

**Figure 5:**
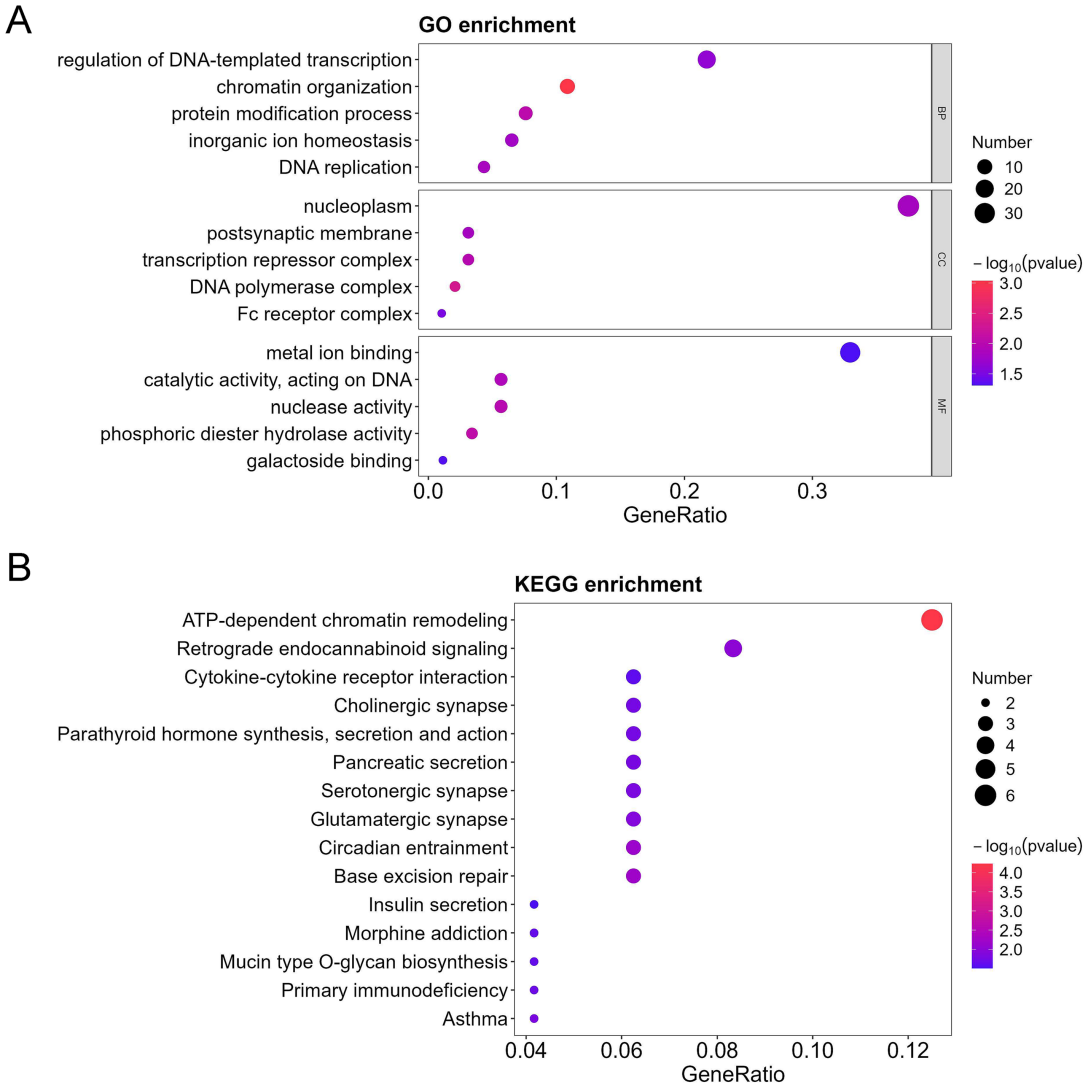
GO, KEGG, and DO analysis of the downregulated differentially expressed proteins (DEPs) in the bronchoalveolar lavage fluid (BALF) of children with mild and severe *Mycoplasma pneumoniae* pneumonia (MPP). **(A)** GO-based enrichment analysis of DEPs (two-sided hypergeometric test, *p* < 0.05), GO terms were sorted by *p* value, and the top 5 terms of each category are displayed. **(B)** KEGG-based enrichment analysis of DEPs (two-sided hypergeometric test, *p* < 0.05), KEGG terms were sorted by *p* value, and the top 15 terms are displayed.

Furthermore, among the 154 DEPs, the 57 upregulated and 97 downregulated DEPs were subjected to GO and KEGG pathway analyses. Immune response, cell cycle, TNF signaling pathway, cytokine-cytokine receptor interaction, and interleukin 17 (IL-17) signaling pathways were mainly enriched in the upregulated proteins (Figures 4A,B). In contrast, the regulation of DNA-templated transcription, chromatin organization, protein modification process, DNA replication in GO, ATP-dependent chromatin remodeling, and cytokine-cytokine receptor interaction were mainly enriched in the downregulated proteins (Figures 5A,B).

### Selection of biomarker combinations for predicting the severity of *Mycoplasma pneumoniae* pneumonia

**Figure 6:**
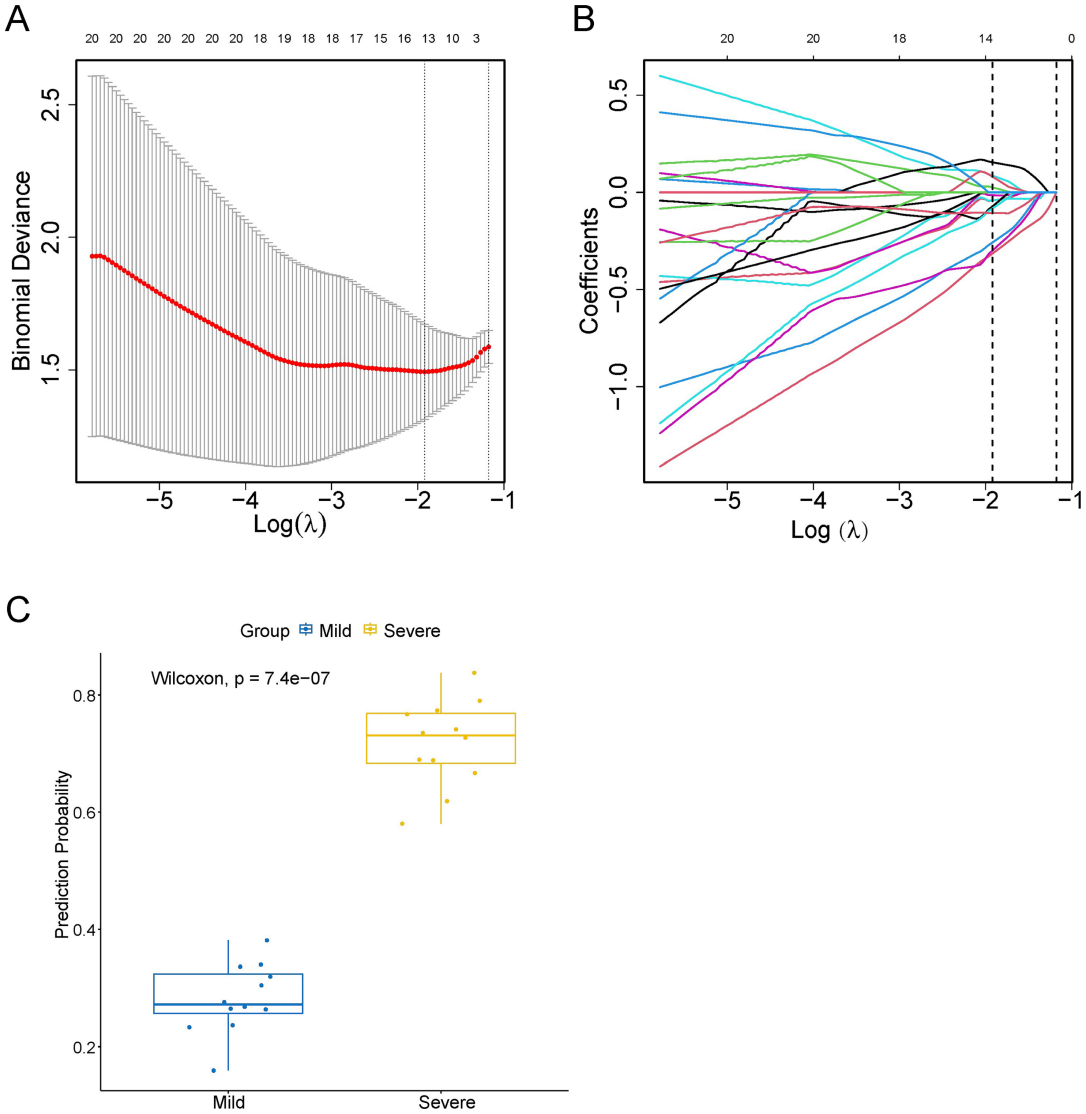
Thirteen proteins detected by screening using least absolute shrinkage and selection operator (LASSO) regression analysis. **(A)** Error Curve. The mean square error (red line) changed with Log(*λ*) during the screening of proteins by the LASSO regression. The upper horizontal axis shows the number of screened proteins. The number of proteins in the minimum mean square error value was the number of proteins screened for subsequent analysis by LASSO regression. **(B)** The coefficient distribution curves of 13 proteins. **(C)** LASSO evaluation figure. The abscissa is the actual value, and the ordinate is the predicted value. The yellow boxplot represents children in the severe MPP group, and the blue boxplot represents children in the mild MPP group.

For variable selection and complexity regularization, LASSO analysis of DEPs was further developed to screen core proteins that were strongly associated with the severity of MPP and provide data for subsequent clinical modeling. Thirteen proteins, including secretory carrier-associated membrane protein 1 (SCAMP1), p21-activated protein kinase-interacting protein 1 (PAK1IP1), tyrosyl-DNA phosphodiesterase 2 (TDP2), glycoprotein-N-acetyl-galactosamine 3-beta-galactosyltransferase 1 (C1GALT1), bromodomain-containing protein 1 (BRD1), cartilage intermediate layer protein 2 (CILP2), protein polybromo-1 (PBRM1), C1GALT1-specific chaperone 1 (C1GALT1C1), Rab proteins geranyl-geranyl transferase component A 1 (CHM), chitinase-3-like protein 2 (CHI3L2), CD209 antigen (CD209), C-X-C motif chemokine 5 (CXCL5) and phosphoserine phosphatase (PSPH) were selected as core proteins, of which, SCAMP1, PAK1IP1, TDP2, C1GALT1, BRD1, CILP2, PBRM1, C1GALT1C1, and CHM were downregulated, and CHI3L2, CD209, CXCL5, and PSPH were upregulated in the severe MPP group (Supplementary Figure S2). The distributions of the binomial deviance and coefficient are shown in Figures 6A,B, respectively. The proteins selected by LASSO clearly distinguished the severity of MPP (Figure 6C).

### Construction of a prediction model of severity of MPP

**Figure 7:**
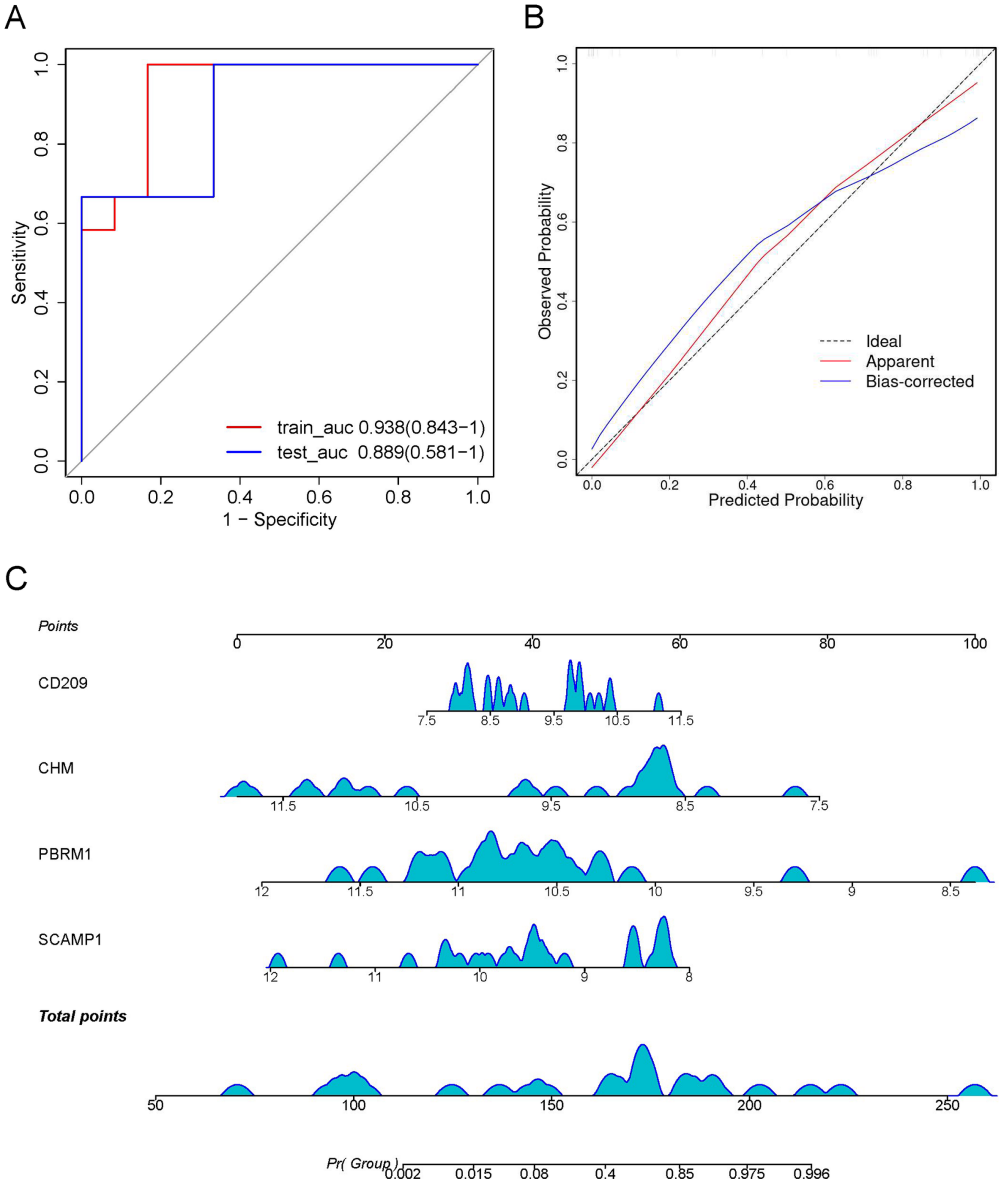
Construction of a visual logistic regression model to predict the severity of *Mycoplasma pneumoniae* pneumonia (MPP). **(A)** Receiver operating characteristic (ROC) curve with optimal area under the curve (AUC) value is shown based on the four differentially expressed proteins (DEPs), SCAMP1, PBRM1, CHM, and CD209, as the best combination. **(B)** Calibration curve showing the predictive value of the model. **(C)** Nomogram for predicting the risk of severe *Mycoplasma pneumoniae* pneumonia (MPP). For each patient, four upward lines were drawn to determine the points received from the four predictors in the nomogram. The sum of these points is located on the “total points” axis. A downward line was then drawn to determine the probability of severe MPP.

Of the 13 DEPs selected by LASSO regression under a random combination assumption, CD209, CHM, PBRM1, and SCAMP1 were identified as the best DEP combination based on the AUC value, and these four proteins were used to develop a clinical risk model. The AUC (95% CI) for the training and test cohorts were 0.938 (0.843–1.000) and 0.889 (0.581–1.000), respectively (Figure 7A). Furthermore, the calibration curve revealed that the model performed well compared with an ideal model (Figure 7B). The severity of MPP was assessed based on the total points from the sum of the four proteins (Figure 7C).

## Discussion

*M. pneumoniae* is an atypical pathogen and one of the most common causes of CAP in children. *M. pneumoniae* used to be considered a benign infection that was usually mild and self-limiting. However, the incidence of severe MPP in children has increased in recent years (Edelstein et al., 2023). Moreover, the increasing emergence of macrolide-resistant *M. pneumoniae* infection in some regions has made treating *M. pneumoniae* infections more difficult (Li et al., 2024; Akashi et al., 2018). The clinical and radiological characteristics of *M. pneumoniae* are often nonspecific, and culture and serological diagnostic tests are insensitive and time-consuming. The use of polymerase chain reaction (PCR) and next-generation sequencing are limited by their high cost and requirement for specialized analyzers make the diagnosis of *M. pneumoniae* infection challenging. Therefore, biomarkers that can be used to diagnose *M. pneumoniae* infection and severe MPP are needed.

Biomarkers detection using body fluids that can be collected using noninvasive methods (e.g., blood, urine) are widely for early diagnosis and predicting the prognosis of various disorders, including cancer, brain disease, and kidney disease (Shama et al., 2023). Previous studies have screened and identified novel potential biomarkers, including proteins and metabolites, in the plasma of children with MPP (Li et al., 2016; Li et al., 2022). Bronchoalveolar lavage (BAL) using routine flexible bronchoscopy is a valuable diagnostic and intervention tool for children with certain respiratory disorders (Gami et al., 2022; Lu et al., 2022). BALF samples can be collected during this procedure. BALF is more useful than other types of body fluid such as blood and urine for studying lung disease because it provides a variety of lung-specific information, including information about immunologic, inflammatory, and infectious processes in the lung parenchyma (Weise et al., 2023; Davidson et al., 2020). In this study, we investigated the proteomic characteristics of BALF in children with MPP using LC–MS/MS. To our knowledge, this is the first study to use high-throughput 4D-DIA proteomic analysis of BALF samples from patients with MPP of different severity. 4D-DIA proteomics adds a fourth dimension of ion mobility to traditional 3D proteomics (retention time, mass-to-charge ratio, and ion intensity). Compared with 3D proteomics, 4D-DIA proteomics improves detection sensitivity, detection depth, and quantitative integrity, and requires a smaller sample size (Meier et al., 2020).

Pulmonary injury caused by *M. pneumoniae* infection has been attributed to the host inflammatory response (Chaudhry et al., 2016). Functional enrichment analysis of the identified DEPs suggested a significant difference in the inflammatory response of children in the mild and severe MPP groups. Cytokines are a broad category of intercellular signaling proteins. By activating cytokine receptor-mediated signaling pathways, cytokines orchestrate diverse immunological processes including providing protection against pathogens and inducing tissue-damaging inflammation (Lin and Leonard, 2019). In this study, cytokine-cytokine receptor interactions were consistently enriched in DEPs. Furthermore, the TNF signaling pathway was enriched in upregulated DEPs. TNF is mainly produced by macrophages (Chen et al., 2023). Alveolar macrophages constitute 80–90% of the cellular components of BALF (de Blic et al., 2000). As the predominant innate immune cells in the distal lung, alveolar macrophages residing within the alveolar lumen act as sentinel cells to encounter incoming pathogens and play a pivotal role in coordinating the activation and resolution of the pulmonary immune responses (Joshi et al., 2018). Therefore, our results suggest that the TNF signaling pathway may be involved in the development of MPP. In addition, the IL-17 signaling pathway was enriched in the upregulated DEPs. The IL-17 family (IL-17A–F) binds to heterodimeric receptors (e.g., IL-17RA/RC complexes) to activate downstream signaling pathways, including NF-κB, MAPKs and C/EBPs, leading to the expression of antimicrobial peptides, cytokines, and chemokines (Gu et al., 2013). Previous reports have shown that the IL-17 signaling pathway is essential for host defense against respiratory pathogens. For example, IL-17RA knockout mice exhibit increased mortality when experimentally infected with intrapulmonary *Klebsiella pneumoniae* (Ye et al., 2001). Impaired IL-17 signaling is associated with host susceptibility to a variety of pathogens including *Streptococcus pneumoniae* (Lu et al., 2008). IL-17 has been reported to plays an important role in respiratory *Mycoplasma* infection and is associated with complications such as pneumonitis and asthma (Luo et al., 2021). This study confirms the essential role of IL-17 and its signaling in the development of MPP.

In this study, we identified four proteins as the best DEP combination. Among them, CD209 was upregulated in the severe MPP group. CD209 is a C-type lectin and is expressed mainly in subsets of dendritic cells and alveolar macrophages (Soilleux et al., 2000; Tailleux et al., 2005). As a component of the innate immune system, CD209 recognizes a wide range of evolutionarily divergent pathogens of public health importance, including *Mycobacterium tuberculosis*, *Streptococcus pneumoniae*, *Klebsiella pneumoniae*, Ebola virus, hepatitis C virus, HIV-1, dengue viruses, and SARS-CoV-2, and consequently activates the immune response (van Kooyk and Geijtenbeek, 2003). CD209 may suppress Toll-like receptor signaling, thereby influencing the Th1/Th2 (proinflammatory/anti-inflammatory) balance and causing *M. tuberculosis*-induced immune suppression, which may be crucial to tuberculosis disease progression (van Kooyk and Geijtenbeek, 2003; Geijtenbeek et al., 2003).

Regarding the other three proteins that were downregulated in the severe MPP group, PBRM1 is a component of the SWI/SNF chromatin-remodeling complex, which targets the complex to specific sites in the genome, recruits additional effector proteins, and alters histone-DNA interactions that control gene expression (Thompson, 2009). PBRM1 is deregulated in tumors and mediates the chronic induction of interferon signaling (Mota et al., 2019; Yamashita et al., 2023). These findings suggest that PBRM1 has multiple functions under physiological and pathological conditions, which require further study.

SCAMP1 plays a pivotal role in the maintenance and regulation of components of the plasma membrane by participating in cellular pathways such as post-Golgi recycling pathways and endosome cell membrane recycling (Castle and Castle, 2005; Law et al., 2012). CHM, a component of the Rab geranyl-geranyl transferase 2 complex, is involved in Rab protein recruitment to vesicles during vesicle trafficking. Therefore, SCAMP1 and CHM may play a role in regulating the growth, survival and invasion of cancer cells (Yun et al., 2017; Song et al., 2017; Goldenring, 2013). Further studies are required to explore the functions and potential mechanisms by which PBRM1, SCAMP1, and CHM influence MPP progression.

This study has some limitations. First, the proteomic analysis was based on a small cohort. Second, there was no control group of children without MPP because obtaining BALF samples from healthy subjects as a control group was not ethically feasible. Therefore, large-sample studies are needed that include BALF from controls with other respiratory conditions such as influenza or respiratory syncytial virus infection.

In conclusion, this study compared proteomic alterations in the BALF of children with mild and severe MPP using 4D-DIA proteomics analysis. The proteomics analysis revealed that the TNF-α and IL-17 signaling pathways may participate in the pathogenesis of MPP by triggering immune and inflammatory responses. Furthermore, the predictive model identified some potentially valuable biomarkers of disease severity, which could potentially also be used as candidates for targeted therapy. The visual logistic model, which includes CD209, PBRM1, SCAMP1, and CHM, can be used to predict severe MPP and these proteins could potentially be used for early diagnosis and clinical intervention in children with MPP.

## Data Availability

The original contributions presented in the study are included in the article/Supplementary material, further inquiries can be directed to the corresponding authors.

## Footnotes

^1^ http://www.geneontology.org/

^2^ https://www.kegg.jp/kegg/
