# Supplementary material for: Proteomic characteristics of bronchoalveolar lavage fluid in children with mild and severe Mycoplasma pneumoniae pneumonia
Source: Front Microbiol. 2025 May 19;16:1595521. doi: 10.3389/fmicb.2025.1595521 (PMC12128088; doi:10.3389/fmicb.2025.1595521)
Supplement: Supplementary file 1 [file Presentation_1.pdf]

Supplementary figures

Fig. S1. Quality control of proteomics experiments. (A) The majority of peptides detected by mass spectrometry were 7–24 amino acids in length. (B) More than 85% of the proteins were polypeptides. (C) Pearson's correlation coefficient analysis and (D) principal components analysis (PCA) were used to assess the quantitative repeatability between duplicate samples and quantitative correlation between different groups of samples.

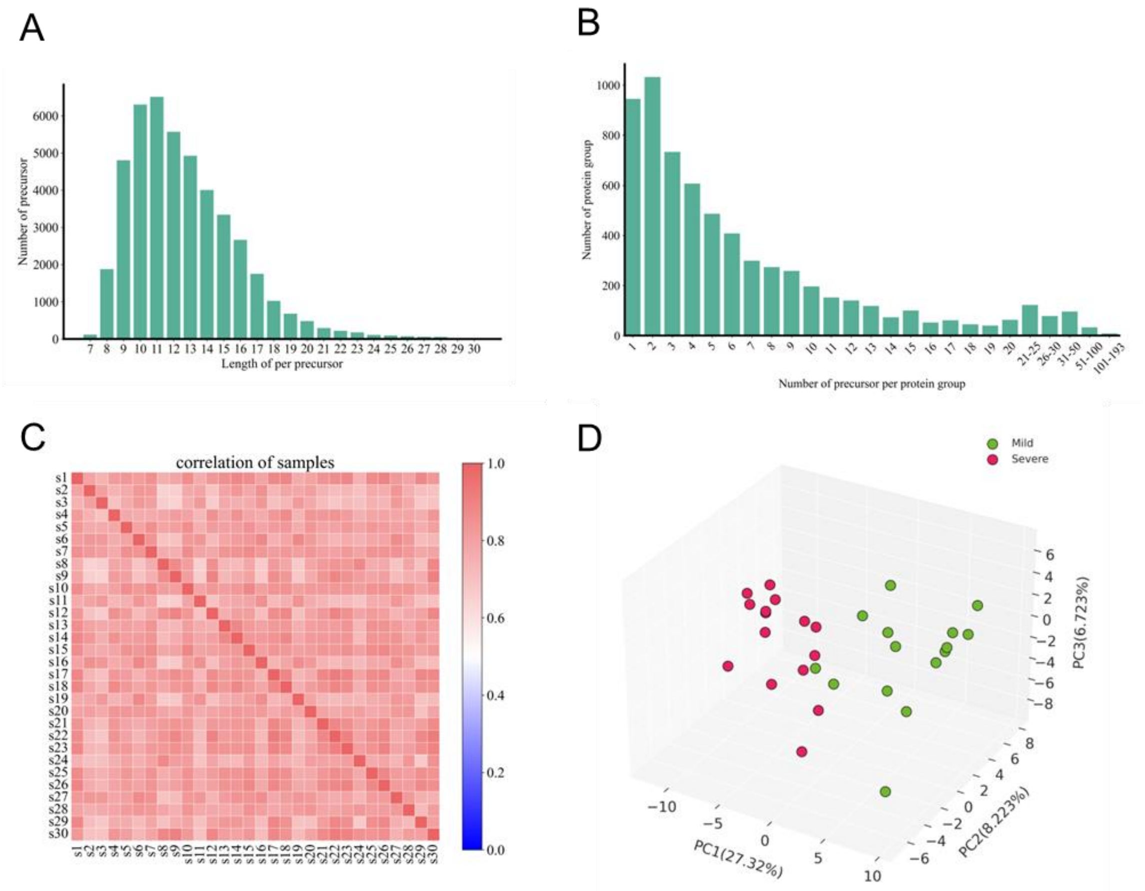

Fig. S2. Comparison of expression of 13 proteins in the bronchoalveolar lavage fluid (BALF) of children with mild and severe *Mycoplasma pneumoniae* pneumonia (MPP) selected using LASSO regression analysis.

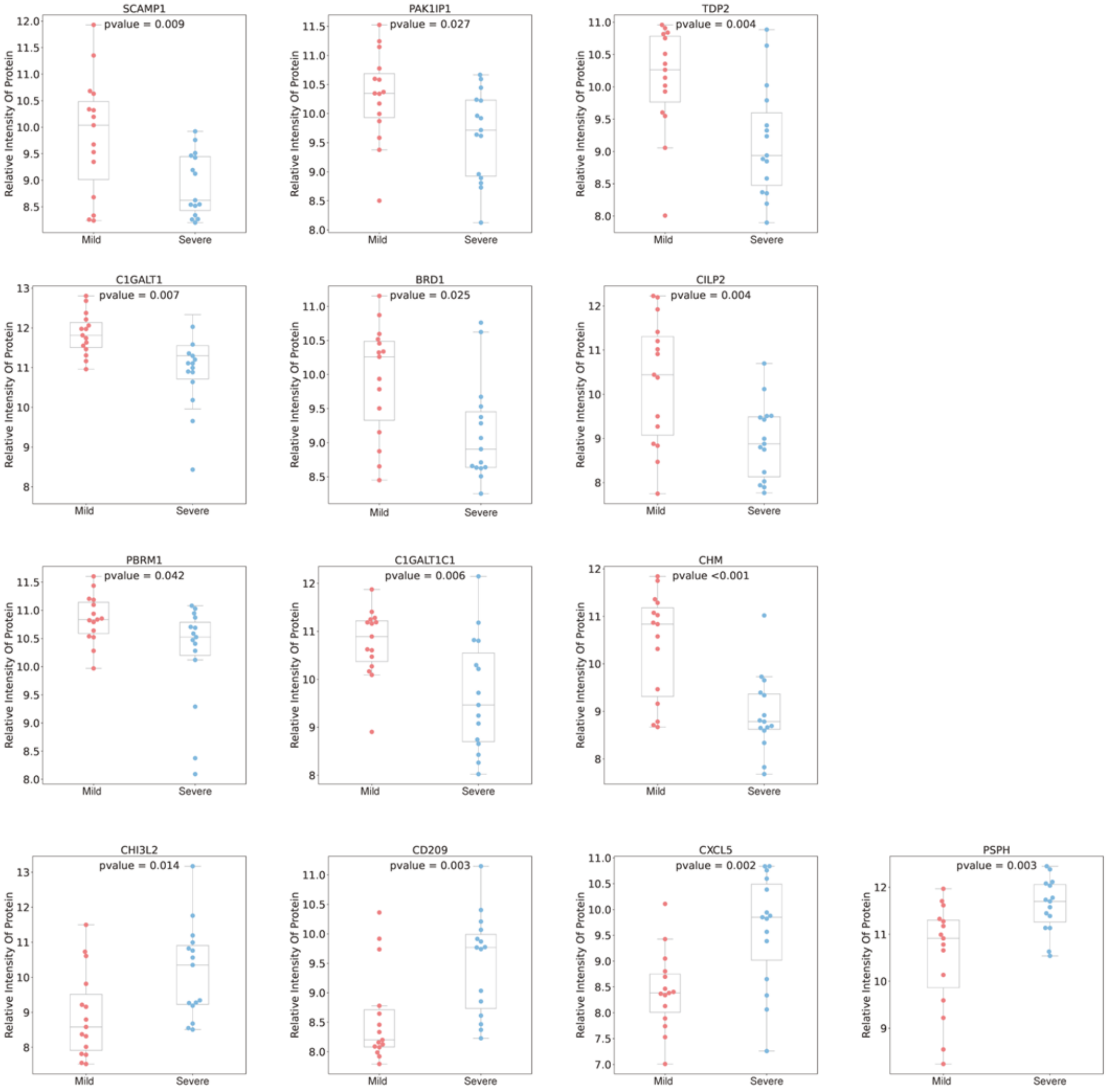

Supplementary information

The mass spectrometry proteomics data have been deposited to the ProteomeXchange Consortium (https://proteomecentral.proteomexchange.org) via the iProX partner repository (Chen et al., 2022; Ma et al., 2019) with the dataset identifier PXD060198.

Chen, T., Ma, J., Liu, Y., Chen, Z., Xiao, N., Lu, Y., Fu, Y., Yang, C., Li, M., Wu, S., *et al.* (2022). iProX in 2021: connecting proteomics data sharing with big data. Nucleic Acids Res *50*, D1522-d1527.

Ma, J., Chen, T., Wu, S., Yang, C., Bai, M., Shu, K., Li, K., Zhang, G., Jin, Z., He, F., *et al.* (2019). iProX: an integrated proteome resource. Nucleic Acids Res *47*, D1211-d1217.
